# Associations between Microglia and Astrocytic Proteins and Tau Biomarkers across the Continuum of Alzheimer’s Disease

**DOI:** 10.3390/ijms25147543

**Published:** 2024-07-09

**Authors:** Julia Doroszkiewicz, Agnieszka Kulczyńska-Przybik, Maciej Dulewicz, Jan Mroczko, Renata Borawska, Agnieszka Słowik, Henrik Zetterberg, Jörg Hanrieder, Kaj Blennow, Barbara Mroczko

**Affiliations:** 1 Department of Neurodegeneration Diagnostics, Medical University of Bialystok, 15-269 Bialystok, Poland; julia.doroszkiewicz@sd.umb.edu.pl (J.D.); agnieszka.kulczynska-przybik@umb.edu.pl (A.K.-P.); mjanek2003@gmail.com (J.M.); renata.borawska@umb.edu.pl (R.B.); 2 Department of Psychiatry and Neurochemistry, Institute of Neuroscience and Physiology, The Sahlgrenska Academy at the University of Gothenburg, 431 80 Mölndal, Sweden; maciej.dulewicz@gu.se (M.D.); henrik.zetterberg@clinchem.gu.se (H.Z.); jorg.hanrieder@neuro.gu.se (J.H.); kaj.blennow@neuro.gu.se (K.B.); 3 Department of Neurology, Jagiellonian University, 30-688 Cracow, Poland; slowik@cm-uj.krakow.pl; 4 Clinical Neurochemistry Laboratory, Sahlgrenska University Hospital, 431 80 Mölndal, Sweden; 5 Department of Neurodegenerative Disease, UCL Institute of Neurology, Queen Square, London WC1N 3BG, UK; 6 UK Dementia Research Institute at UCL, London WC1N 3AR, UK; 7 Hong Kong Center for Neurodegenerative Diseases, Clear Water Bay, Hong Kong, China; 8 Wisconsin Alzheimer’s Disease Research Center, University of Wisconsin School of Medicine and Public Health, University of Wisconsin-Madison, Madison, WI 53792-2460, USA; 9 SciLifeLab, University of Gothenburg, 405 30 Gothenburg, Sweden; 10 Department of Biochemical Diagnostics, Medical University of Bialystok, 15-269 Bialystok, Poland

**Keywords:** Alzheimer’s disease, microglia, astrocytes, NGAL, CXCL-11, sTREM1, sTREM2, neuroinflammation

## Abstract

Recent investigations implicate neuroinflammatory changes, including astrocyte and microglia activation, as crucial in the progression of Alzheimer’s disease (AD) Thus, we compared selected proteins reflecting neuroinflammatory processes to establish their connection to AD pathologies. Our study, encompassing 80 subjects with (*n* = 42) AD, (*n* = 18) mild cognitive impairment (MCI) and (*n* = 20) non-demented controls compares the clinical potential of tested molecules. Using antibody-based methods, we assessed concentrations of NGAL, CXCL-11, sTREM1, and sTREM2 in cerebrospinal fluid (CSF). Proinflammatory proteins, NGAL, and CXCL-11 reached a peak in the early stage of the disease and allowed for the identification of patients with MCI. Furthermore, the concentration of the anti-inflammatory molecule sTREM2 was highest in the more advanced stage of the disease and permitted differentiation between AD and non-demented controls. Additionally, sTREM2 was biochemically linked to tau and pTau in the AD group. Notably, NGAL demonstrated superior diagnostic performance compared to classical AD biomarkers in discriminating MCI patients from controls. These findings suggest that proteins secreted mainly through microglia dysfunction might play not only a detrimental but also a protective role in the development of AD pathology.

## 1. Introduction

Alzheimer’s disease (AD) is a prevalent and debilitating illness that primarlily affects older individuals. It is characterized by memory loss, aphasia, and serious problems with short- and long-term memory [1]. The accumulation of amyloid β (Aβ) fibrils as well as the presence of insoluble plaques, neurofibrillary tangles (NFT) composed of hyperphosphorylated tau, loss of neurons and synapses, and atrophy of memory-related brain areas are the most common features of AD [2]. The buildup and aggregation of Aβ1-42 initiates a series of pathological processes, including neuroinflammation, cytoskeletal abnormalities, synaptic and neuronal network dysfunction, and ultimately neuronal cell death [3,4,5]. The illness progresses gradually; it may take up to 15–25 years for symptoms to develop [1].

Inflammation in the peripheral and central nervous systems (CNS) may contribute to AD pathology. Astrocytes and microglia, along with some peripheral immune cells that infiltrate the brain, contribute to neuroinflammation [6,7]. While neuroinflammatory processes can have important neuroprotective roles, persistent and escalating neuroinflammation can have negative consequences on brain function leading to neurological impairment and neurodegeneration [8]. Microglial cells are involved in both innate and adaptive immune responses to pathogens. Moreover, it was established that the type of microglia is crucial for the regulation of myelination and neurogenesis [6,9]. In pathological conditions, microglial cells become activated and secret various inflammatory proteins. Similarly, astrocytes could be activated by pathogenic Aβ, tau species, and proinflammatory cytokines and then release the latter [10,11]. By continuously releasing pro-inflammatory cytokines, chronically activated microglia and astrocytes can cause brain injury by making neurons more susceptible to cell death and encouraging the creation of dangerous protein aggregates [6,12,13]. Some researchers postulate that more intricate interaction between cells of innate immunity and proteinopathy is associated with neurodegeneration. Therefore, studies concerning inflammatory dysregulation mechanisms in neurodegenerative dementias are especially important to deep knowledge about their contribution to the development of the diseases. In our paper, we investigated selected pro- and anti-inflammatory proteins secreted by activated microglia and astrocytes in the continuum of the disease to assess the relationship between tested molecules and the main pathological indicators of dementia disease, particularly tau pathology. Most investigations concerning inflammatory indicators in AD are focused on amyloid pathology; thus, studies referring to relationships between tau pathology and inflammation in the CNS are also needed. The preclinical studies suggest that inflammation may induce tau hyperphosphorylation at both pre- and post-tangles periods [14,15]. Furthermore, recent findings reported that microglia dysfunction could influence tau phosphorylation, synaptic loss, as well as memory impairment even at the very early stages of the disease, before β-amyloid positivity. Considering that findings from similar studies offer a promising candidate therapeutic target to halt cognitive decline associated with aging and AD, further investigations are necessary [16].

The literature evidence indicates that neutrophil gelatinase-associated lipocalin (NGAL) also known as lipocalin-2 (LCN2), or siderocalin is one of the proteins secreted by activated microglia and astrocytes in AD. NGAL is a member of the diverse lipocalin family of carriers of lipophilic/hydrophobic molecules [17]. While NGAL primarily originates from neutrophils, its expression was also discovered in various other cells including tubular cells in the kidney, heart, lung, and dendritic cells [18]. Some research suggests that lipocalin may affect several neurobiological processes, including inflammation, signaling for cell death and survival, as well as iron metabolism. In the CNS, NGAL specifically induces insulin resistance, activates gliosis, and causes neuronal death [19,20,21]. NGAL may facilitate the infiltration of neutrophils and macrophages into the brain and stimulate pro-inflammatory activation of glial cells [22]. Dekens et al. described elevated levels of NGAL in the hippocampus and amygdala of AD patients. Furthermore, the authors showed colocalization of NGAL with microglia and neurons [23]. The study by Llorens et al. revealed that this protein allows for the discrimination of vascular dementia (VaD) from AD without coexisting vascular changes with high accuracy [24].

Another protein secreted by activated microglia is a small member of the CXC chemokine family—CXCL11. It was first discovered in mouse astrocytes treated with interferon beta (IFN-β). It has been shown that human astrocytes and fetal human microglia may be stimulated to produce the CXCL11 protein in response to IFN-Y alone or in combination with interleukin (IL)-1 [25]. It is also highly expressed by elements of the gastrointestinal system such as the liver, pancreas and in lower amounts expressed in the small intestine [26]. Elevated CXCL11 was found in subcutaneously infected mouse brains and the cerebrospinal fluid of patients with neuro-inflammatory illnesses such as bacterial meningitis and viral meningitis [27,28]

In the available literature, there is a lack of data concerning CSF levels of soluble triggering receptor expressed on myeloid cells-1 (sTREM1) and CXCL-11, inflammatory proteins released from neutrophils, microglia, and astrocytes in AD-associated neuropathological processes. TREM1 belongs to the immunoglobulin superfamily and is primarily provided by microglia, myeloid cells including neutrophils, macrophages, and monocytes [29]. TREM1 plays a significant role in the induction and exacerbation of inflammatory responses also in the CNS. Some studies reported that it is implicated in the development of numerous infectious and non-infectious diseases, including autoimmune diseases, malignancies, and neurodegenerative diseases [30,31]. However, still little is known about its role in AD.

While many studies describe that TREM1 promotes neuroinflammation, TREM2 is known as the inhibitor of this state [31]. Triggering receptor expressed on myeloid cells-2 (TREM2) is considered a neuroprotective factor in the CNS. It is produced by microglia cells. Furthermore, it has been proposed that sTREM2 may increase TREM2 protein synthesis and microglial survival by stimulating the synthesis of innate immune components [32]. Even though there is growing evidence that AD is associated with neuroinflammation, more research is needed to understand the relationship between these mechanisms and the pathologies of tau and Aβ, as well as whether these relationships matter more early in the illness or later on.

Therefore, the primary aim of this study was to assess selected pro- and anti-inflammatory proteins reflecting microglial and astrocytic activation and compare their levels with cognitive impairment in patients with AD and MCI. Secondly, the current study determined the association of CSF concentrations of tested biomarkers with the main mechanisms of amyloid and tau pathologies. This is the continuation of our previous works [33,34]. However, it is worth noting that investigations on the clinical potential use of astrocytic and microglial indicators of inflammatory state in the continuum of the disease are particularly crucial not only for diagnostic but also for therapeutic purposes. Additionally, according to our knowledge, this is the first study that investigates the concentrations of CXCL11 in CSF of AD patients, especially in correlation with microglia/astrocytic indicators and classical biomarkers of AD.

## 2. Results

### 2.1. Patient Characteristics and Comparison of CSF Concentrations of Tested Proteins Related to Inflammation

The summary of the CSF classical biomarker values in the examined groups was presented in Table 1. AD biomarkers were assesed in CSF samples of all patients. Statistical analysis revealed statistically significant differences between all the study groups for CSF concentrations of NGAL (*p* < 0.001, χ^2^ = 15.71), CXCL11 (*p* ≤ 0.001, χ^2^ = 39.26), sTREM1 (*p* = 0.001, χ^2^ = 13.20), sTREM2 (*p* = 0.037), Aβ1-42 (*p* < 0.001, χ^2^ = 22.94), Aβ1-42/Aβ1-40 ratio (*p* < 0.001, χ^2^ = 38.83), tau (*p* < 0.001, χ^2^ = 48.97) and pTau181 (*p* < 0.001, χ^2^ = 43.25) (Table 1 and Table 2).

The NGAL levels in CSF differed significantly between the patients with AD as well as the controls and also between the MCI patients and controls. The highest CSF concentration of NGAL was observed in the group of patients with AD in comparison to CTRL (*p* < 0.01). sTREM2 concentrations were statistically significantly higher in the AD group vs. CTRL, similar to the MCI group vs. CTRL. The highest levels of CXCL11 were discovered in the MCI group, followed by the AD group, with the lowest in the CTRL group. Interestingly, there was a significant statistical difference between the AD and MCI groups.

**Figure 1:**
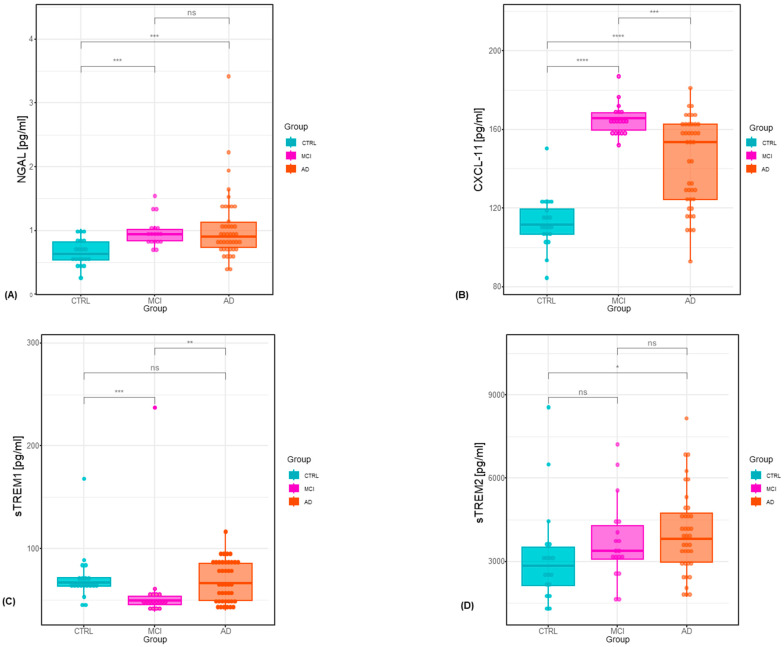
(**A**) Cerebrospinal fluid neutrophil gelatinase-associated lipocalin concentrations by group. (**B**) Cerebrospinal fluid CXCL-11 concentrations by group. (**C**) Cerebrospinal fluid soluble triggering receptor expressed on myeloid cells-1 concentrations by group. (**D**) Cerebrospinal fluid soluble triggering receptor expressed on myeloid cells-2 concentrations by group; CTRL—control; MCI—mild cognitive impairment; AD—Alzheimer’s disease. **** *p* < 0.0001, *** *p* < 0.001, ** *p* < 0.01, * *p* < 0.05, ns—not significant.

A significantly higher concentration of CXCL11 was found in the AD and MCI patients in comparison to controls. CXCL11 concentrations in the MCI group were significantly higher than in AD. sTREM1 concentrations in CSF differed significantly between patients with MCI and controls, as well as also between the MCI patients and the AD subjects (Table 2, Figure 1).

### 2.2. Association between Tested Pro- and Anti-Inflammatory Molecules and Classical Biomarkers

**Figure 2:**
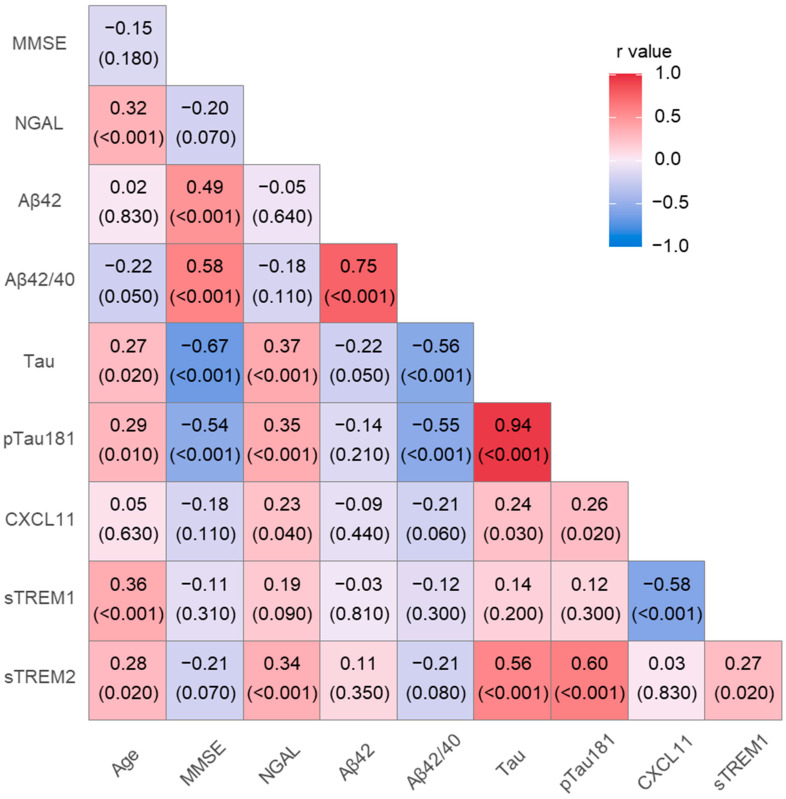
Cerebrospinal fluid levels of pro and anti-inflammatory proteins in the whole study group. NGAL—neutrophil gelatinase-associated lipocalin; sTREM1—soluble triggering receptor expressed on myeloid cells-1; sTREM2—soluble triggering receptor expressed on myeloid cells-2; *p* < 0.001.

The associations between levels of pro- and anti-inflammatory proteins and classical AD biomarkers were analyzed using the Spearman rank correlation test. In the entire study population, significant positive correlations were observed between CSF levels of NGAL and tau (R = 0.37, *p* < 0.001), pTau (R = 0.35, *p* < 0.001), age (R = 0.32, *p* < 0.001), CXCL11 (R = 0.23, *p* = 0.04) and sTREM2 (R = 0.34, *p* < 0.001) (Figure 2). Levels of sTREM2 correlated positively with sTREM1 (R = 0.27, *p* = 0.02), tau (R = 0.56, *p* < 0.001), pTau (R = 0.6, *p* < 0.001), age (R = 0.26, *p* = 0.02), while sTREM1 showed a positive significant correlation with age (R = 0.36, *p* < 0.001) and a negative correlation with CXCL11 (R = −0.58, *p* < 0.001). CXCL-11 and tau (R = 0.26, *p* = 0.02) also showed a positive correlation.

**Figure 3:**
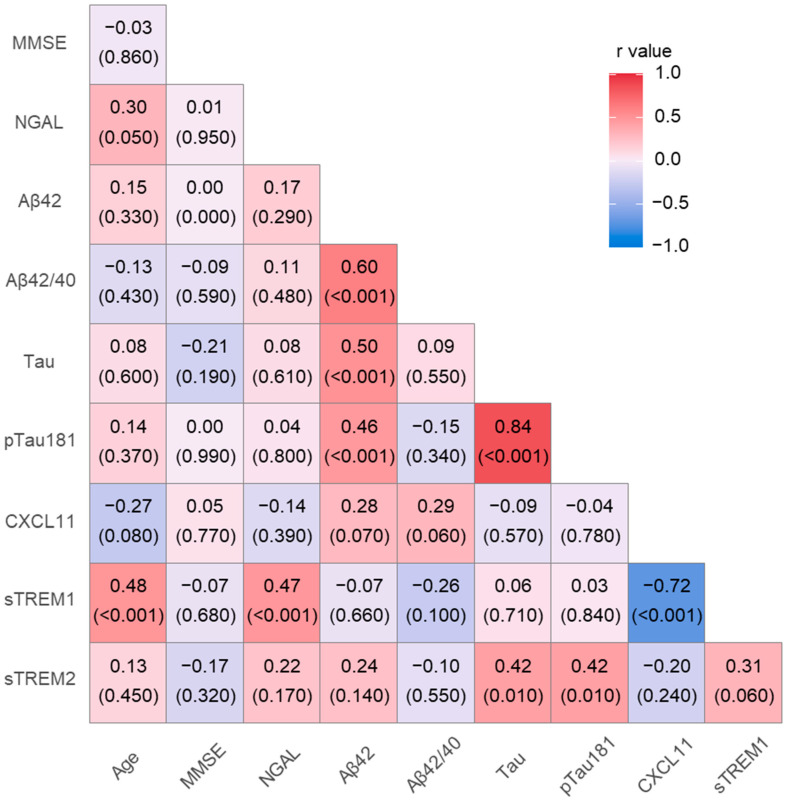
Cerebrospinal fluid levels of pro and anti-inflammatory in AD group. NGAL—neutrophil gelatinase-associated lipocalin; sTREM1—soluble triggering receptor expressed on myeloid cells-1; sTREM2—soluble triggering receptor expressed on myeloid cells-2.

In AD patients, the CSF NGAL level was significantly associated with sTREM1 (R = 0.47, *p* < 0.001) concentrations and age (R = 0.32, *p* < 0.001). Additionally, sTREM2 correlated positively with tau (R = 0.42, *p* = 0.01) and pTau (R = 0.42, *p* = 0.01). Moreover, sTREM1 correlated with age (R = 0.48, *p* < 0.001) (Figure 3).

In the MCI group, no significant correlations with NGAL or other proteins were observed. However, there was a strong, significant correlation between sTREM2 and tau (R = 0.71, *p* < 0.001) and pTau (R = 0.77, *p* < 0.001) (Figure S1 in Supplementary Materials).

### 2.3. Diagnostic Usefulness of Candidate Biomarkers

**Figure 4:**
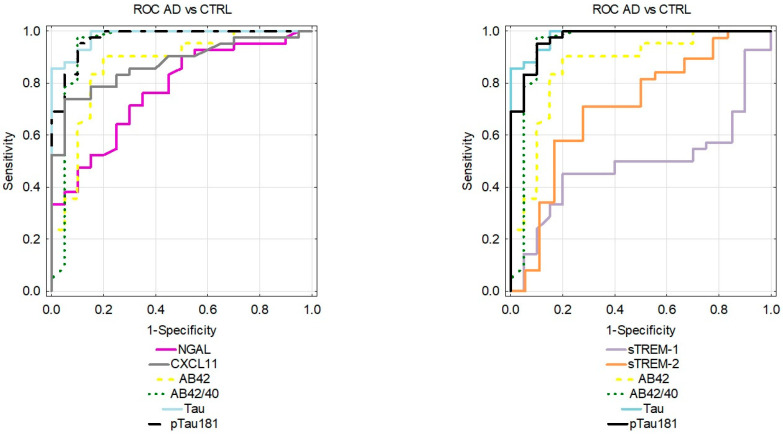
Comparison of area under ROC curves (AUC) for cerebrospinal fluid levels of pro and anti-inflammatory and classical AD biomarkers in AD and CTRL groups. NGAL—neutrophil gelatinase-associated lipocalin; sTREM1—soluble triggering receptor expressed on myeloid cells-1; sTREM2—soluble triggering receptor expressed on myeloid cells-2. AD—Alzheimer’s disease; CTRL—control.

**Figure 5:**
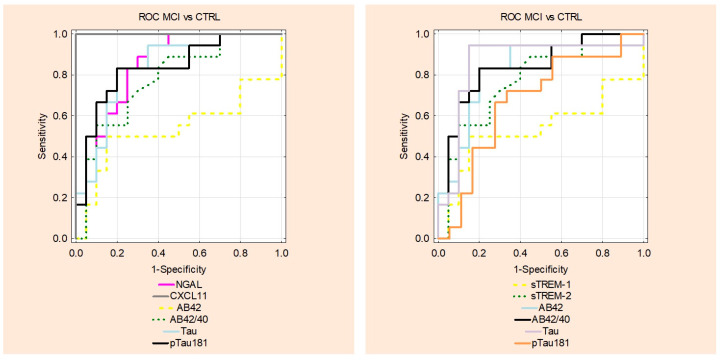
Comparison of area under ROC curves (AUC) for cerebrospinal fluid levels of pro and anti-inflammatory and classical AD biomarkers in MCI and CTRL groups. NGAL—neutrophil gelatinase-associated lipocalin; sTREM1—soluble triggering receptor expressed on myeloid cells-1; sTREM2—soluble triggering receptor expressed on myeloid cells-2; MCI—mild cognitive impairment; CTRL—control.

The analysis of the receiver operating characteristic curves (ROCs) was performed for the differentiation between the AD and MCI groups and the CTRL group. The proinflammatory proteins showed the following results: NGAL (AUC = 0.773, *p* < 0.001), CXCL-11 (AUC = 0.875, *p* < 0.001) while differentiating between the AD and CTRL groups. On the other hand, anti-inflammatory protein depicted the following data: sTREM2 (AUC = 0.705, *p* = 0.01) in differentiating between the AD and CTRL groups; however, they were not better than classical biomarkers of AD (Figure 4). In the MCI group, diagnostic performance of the sTREM1 (AUC = 0.858, *p* < 0.001) and NGAL (AUC = 0.844, *p* < 0.001) was better than established biomarkers such as Aβ1-42 (AUC = 0.536, *p* = 0.723), Aβ1-42/1-40 ratio (AUC = 0.778, *p* < 0.001), tau (AUC = 0.836, *p* < 0.001), and pTau (AUC = 0.8363, *p* < 0.001) (Figure 5). The ROC for the AD vs. MCI group can be seen in Supplementary Materials (Figure S2).

## 3. Discussion

The chronic activation of microglia and astrocytes in response to misfolded and aggregated proteins leading to inflammation affects the course and severity of the disease in AD. That state is typical of the injured parts of the central nervous system (CNS). Neurotoxicity and brain damage in AD are primarily caused by gliosis and inflammatory mechanisms, which can start before the onset of the disease or develop alongside degenerative changes (such as neuronal loss, synaptic loss, and neurofibrillary tangle formation) as the disease advances. Astrogliosis was noted in AD characterized by increased levels of glial fibrillary acidic protein (GFAP) which has been observed in AD, particularly in cases with extended disease duration. [35,36]. Several inflammation-related proteins can be observed in the CSF of AD patients. Recent studies have highlighted the potential connection between proinflammatory cytokine levels and cognitive decline in individuals with mild cognitive impairment (MCI) and AD. Therefore, it is crucial to investigate the proteins that reflect neuroinflammation as potential indicators of the development of cognitive deficits and the course of the illness, particularly in light of the lack of efficacy of therapeutic approaches that target amyloid plaques and neurofibrillary tangles. Considering the aforementioned facts, this study aimed to evaluate the levels of selected pro and anti-inflammatory proteins secreted by activated astrocytes and/or microglia in AD continuum patients and individuals without cognitive decline to investigate their relationship with amyloid and tau pathology in different stages of the dementia process.

Our findings are consistent with other studies and support the upregulation of both proinflammatory and anti-inflammatory molecules released by microglia and astrocytes into the CSF of patients with varying degrees of dementia. The present study specifically found that among all tested proteins, the highest concentration in the earliest phases of the disease, in the MCI patients, were detected for CXCL11 and NGAL. Interestingly, in more severe stages, the level of CXCL11 was slightly lower. According to our knowledge, this is the first study that investigates the concentrations of CXCL11 in CSF of AD patients. However, elevated levels of CXCL11 in cerebrospinal fluid have been shown in patients with some neuro-inflammatory diseases such as bacterial and viral meningitis [28]. Additionally, studies on animal models revealed increased expression of CXCL11 after ischemic damage in the cortex. The authors postulated that activated microglia is a primary source of CXCL11 expression [37]. Our findings indicate that increased levels of CXCL11 in patients with MCI could be a part of the initial immunological response activated in microglia and become an inflammatory process in the brain. At more advanced stages of the disease, this process seems to be alleviated which reflects lower levels of the protein in fully developed AD. However, extensive research is still needed.

In accordance with our findings, a recent study by das Neves et al. showed higher concentrations of CSF NGAL in patients with AD and MCI [38]. Overexpression of NGAL was also found in the brains of AD patients in comparison to controls in areas of the brain that are affected by AD, such as the pre-frontal cortex, amygdala, and hippocampal regions. However, there are also conflicting studies that describe lower concentrations of NGAL in AD vs. CTRL [23,39], and MCI vs. CTRL [39,40]. What is more, a recent meta-analysis showed no differences in AD vs. CTRL but also MCI vs. CTRL [41]. In our opinion, higher concentrations of NGAL in CSF during disease progression may be associated with the increasing activity of astrocytes and neutrophils participating in the inflammatory response. Additionally, given that the blood–CSF barrier in our patients was achieved, our results suggest that higher CSF NGAL levels may reflect pathological processes in the CNS, not the systemic ones. There is an agreement that astrocytes are thought to be the primary producers of NGAL in the brain. This potential function for NGAL in maintaining neuronal homeostasis relates to iron transport, by being able to deliver iron through a transferrin-independent mechanism [42,43,44]. Additionally, NGAL tends to drive the upregulation of iron-related and proinflammatory genes in astrocytes in response to Aβ1-42, which favors the neurotoxic phenotype [45,46]. Interestingly, NGAL is secreted also by neutrophils at sites of infection and choroid plexus (in which most of the CSF is produced and secreted) epithelial cells, where it acts as an acute phase protein [47,48]. Moreover, recent studies show that NGAL is also linked to changes in overall behavior, cognitive functions, and depression [49,50]. However, Ferreira et al. described the potential neuroprotective roles of the NGAL as regulating the balance between pro and anti-inflammatory responses [44]. As a component of the acute-phase response, LCN2 functions in the initial stages of antimicrobial defense to sequester bacterial siderophores—bacterial compounds that have a greater affinity for iron than the host’s iron-binding proteins [51]. Other authors suggested that increased CSF NGAL levels in AD patients might be connected with the altered secretory activity of damaged choroid plexus in patients with advanced stages of AD [52,53]. Furthermore, our study revealed that tested proinflammatory proteins showed positive correlations only in the advanced stages of dementia. In the AD group, NGAL and sTREM1 showed positive correlations with themselves.

Findings from our investigation suggest that some protective mechanisms in more advanced stages are activated. In more advanced stages of dementia, the accelerated activity of protective mechanisms in the brain could reflect elevated levels of the anti-inflammatory protein sTREM2. Our results are in agreement with other studies that described increased concentrations of sTREM2 in the CSF of AD patients [54]. In addition, we postulate that sTREM2 could be one of the molecular indicators of activated neuropathological mechanisms in Alzheimer’s disease.

We evaluated the relationships between proteins associated with inflammation and classical biomarkers. Among all tested inflammatory biomarkers only sTREM2 showed correlation with tau and pTau in AD patients. Interestingly, this correlation between sTREM2 and tau proteins was observed even in at the early stages of the disease, specifically in the MCI group. Our findings suggest that an increase in the concentration of anti-inflammatory proteins may be linked to the activation of the immune response to the intrathecal synthesis of the misfolded proteins, acting as a protective mechanism. It is important to note that this correlation is stronger in the earlier stages of the disease but also maintains in patients with fully developed AD. Higher levels of sTREM2 suggest microglia activation and might be confirming undergoing neurodegeneration. The results are in agreement with other authors [55,56]. A study on a hTau (human MAPT expressed but not endogenous mouse Mapt) mouse model revealed that TREM2 deficiency exacerbates tau phosphorylation and aggregation throughout the early stages of the disease. Additionally, a recent study described that microglia-derived sTREM2 selectively binds to transeglin 2 and inhibits the RhoA/ROCK/GSK3β signaling pathway thereby reducing tau hyperphosphorylation [57].

Furthermore, we assesed the diagnostic usefulness of the tested proteins based on the AUC results. The best discriminatory capability was demonstrated for sTREM1 and NGAL in the MCI group, better than other tested proteins, including established Alzheimer’s biomarkers. While discriminating between the AD and CTRL, results were also high, especially for NGAL although the AUC values did not surpass those of the classical biomarkers. This might suggest that these proteins might be most effective in the early stages of neurodegeneration.

Understanding inflammatory pathomechanism may allow to restrict AD pathology and may open avenues for novel diagnostics tools, as well as therapeutic targets. Findings from our study suggest that harmful action of some proinflammatory molecules microglia and astrocytic activation-mediated may participate in the progression of the disease at the early stages. However, we acknowledge that our study has limitations in terms of the study cohort population. Nevertheless, in the later stages of the disease anti-inflammatory proteins, including sTREM2, appear to play a crucial role as mechanisms against the development of hyperphosphorylated tau pathology.

## 4. Materials and Methods

### 4.1. Material

The study population consisted of 80 subjects (*n* = 55 women, *n* = 25 men; median age: 74 (63.3–80)) recruited at the Department of Neurology, Jagiellonian University Hospital, Krakow, Poland, and included 42 AD patients (*n* = 33 women, *n* = 9 men; age: 75.5 (64–80)), 18 subjects with MCI (*n* = 11 women, *n* = 7 men; age: 75.5 (70.3–78)), and 20 non-demented controls (*n* = 12 women, *n* = 8 men; age: 68 (63.3–76.8)). The Bialystok University study (No. R-I-002/103/2019) was approved by the Ethics Committee, and the research was carried out in accordance with the Declaration of Helsinki in the Department of Neurodegeneration Diagnostics at the Medical University of Bialystok. Prior to any procedures, each patient signed an informed consent form. The clinical diagnosis of the research groups involved the use of standard medical, physical, and neurological examinations, laboratory screening tests, a neurocognitive assessment, and brain computed tomography or magnetic resonance imaging. Cases of Alzheimer’s disease with sporadic occurrences formed the AD group. Throughout their medical interview, none of the study’s patients disclosed a family history of Alzheimer’s. The diagnosis of AD has been determined using the National Institute on Aging and Alzheimer’s Association (NIA-AA) criteria [58,59]. To provide the most accurate clinical diagnosis of AD, neurochemical data (levels of Aβ1-42, tau, and pTau181 as well as values of the Aβ1-42/Aβ1-40 ratio) were combined with neuroimaging and neuropsychological tests. The MMSE score (range 0–30) was used to determine dementia severity (AD patients (MMSE: 22 (19–24)), MCI patients (MMSE: 27.5 (26–29)), and controls (MMSE: 28.1 (27–30))). Patients from whole study group (AD, MCI and CTRL) with raised albumin quotients (QAlb), a sign of dysfunction in the blood–CSF barrier, abnormalities in CT or MRI scans, and suspected cerebrovascular disorders (such as cerebral hemorrhage, aortic aneurysm, intracranial aneurysm, stroke, or arteriovenous malformation) and also with visible signs of blood in CSF were excluded from the study.

The control group comprised individuals who were not experiencing subjective memory impairments and did not meet the MCI criteria but who might experience recurring headaches. None of the patients in this group displayed any meaningful changes in the levels of the recognized biomarkers for AD (Aβ1-42, tau, and pTau181), which allowed the exclusion of the symptoms’ organic background. An Erlangen Score of 0 points across all 18 of the participants in this group supported these findings.

### 4.2. Biochemical Measurements

CSF samples were taken using lumbar punctures in the L3/L4 or L4/L5 interspace and transferred into polypropylene tubes. All CSF samples were frozen at −80 °C, aliquoted, and centrifuged before analysis. The concentrations of analyzed proteins (NGAL, CXCL11, sTREM1, sTREM2, Aβ1-42, Aβ1-40, tau, and pTau181) in the CSF were measured in the Department of Neurodegeneration Diagnostics, Medical University of Bialystok, Poland.

Neurochemical dementia diagnostics (NDD) biomarker concentrations were measured using IBL kits (RE59661, RE59651, Hamburg, Germany) for Aβ1-42 and Aβ1-40 and Fujirebio kits (81572, 81574, Gent, Belgium) for tau and pTau181 proteins.

R&D Systems, Abingdon, UK, supplied the ELISA kits that were used for the NGAL analysis. Luminex Human Discovery assay plates from R&D Systems, Abingdon, UK, and a Luminex 200 analyzer (multiplexing, multiparametric, fluorescence laser reading system on microspheres for the simultaneous determination of multiple parameters) were used for the analysis of CXCL11, sTREM1, and sTREM2. For every standard, control, and sample duplicate measurements were evaluated in accordance with the manufacturer’s protocols.

### 4.3. Statistical Analysis

The PMCMRplus package in the statistical software RStudio (Version 1.4.1106, Boston, MA, USA) and Statistica 13.3 (StatSoft Polska, Krakow, Poland) were used to perform nonparametric tests. The Shapiro–Wilk test demonstrated that the protein concentrations were not distributed normally. The comparisons between the AD, MCI, and control groups were performed using the Kruskal–Wallis test. The post hoc Dwass–Steele–Critchlow–Fligner test was then used to assess significant differences between the levels of the tested groups to determine which groups had statistically significant differences. The results are presented as medians and interquartile ranges. Statistical significance was set at *p* < 0.05. In addition, the receiver operating characteristic (ROC) curve and area under curve (AUC) analysis was used to determine the diagnostic usefulness of the tested proteins as potential neuroinflammation-related biomarkers for AD.

## Figures and Tables

**Table 1:** Concentrations of tested classical biomarkers in the study group.

Tested Variables	Median (Interquartile Range)			*p* (Kruskal–Wallis Test)
	AD	MCI	Controls	
Group size (F/M)	42 (33/9)	18 (11/7)	20 (12/8)	
Age	75.5 (64–80)	75.5 (70.3–78)	68 (63.3–76.8)	
MMSE	22 (19–24)	27.5 (26–29)	28.1 (27–30)	
Aβ1-42 (pg/mL)	502.7 (381–666)	802 (475–1045)	895 (792–1000)	<0.001
Aβ1-42/1-40 ratio	0.033 (0.029–0.04)	0.045 (0.0365–0.058)	0.066 (0.055–0.076)	<0.001
tau (pg/mL)	669 (576–897)	389 (327–495)	223 (192–273)	<0.001
pTau181 (pg/mL)	83.2 (72.7–109)	57.2 (46.9–68.41)	37.5 (33.4–42.2)	<0.001

AD—Alzheimer’s disease; MCI—mild cognitive impairment; Aβ—amyloid β; F—Female; M—Male.

**Table 2:** Concentrations of tested proteins connected to the neuroinflammation in the study group.

Tested Variables	Median (Interquartile Range)			*p* (Kruskal–Wallis Test)	*p* (Dwass–Steele–Critchlow–Flinger Test)		
	AD	MCI	Controls		AD vs. CTRL	AD vs. MCI	MCI vs. CTRL
NGAL (pg/mL)	0.907 (0.739–1.13)	0.945 (0.841–1.01)	0.629 (0.538–0.822)	<0.001	<0.01	0.908	<0.001
CXCL11 (pg/mL)	154 (124–163)	166 (160–168)	111 (107–120)	<0.001	<0.001	0.002	<0.001
sTREM1 (pg/mL)	66 (49.5–85.2)	50 (45.2–53.3)	67 (63.2–71.4)	0.001	0.989	0.008	<0.001
sTREM2 (pg/mL)	3805 (2968–4732)	3376 (3081–4279)	2835 (2124–3516)	0.037	0.037	0.699	0.179

NGAL—neutrophil gelatinase-associated lipocalin; sTREM1—soluble triggering receptor expressed on myeloid cells-1; sTREM2—soluble triggering receptor expressed on myeloid cells-2; AD—Alzheimer’s disease; MCI—mild cognitive impairment; CTRL—control.

## Data Availability

The original data presented in the study are openly available this publication.

## Supplementary Materials

The following supporting information can be downloaded at: https://www.mdpi.com/article/10.3390/ijms25147543/s1.

## References

[1] Scheltens P., De Strooper B., Kivipelto M., Holstege H., Chételat G., Teunissen C.E., Cummings J., van der Flier W.M. (2021). Alzheimer’s Disease. Lancet.

[2] El Kadmiri N., Said N., Slassi I., El Moutawakil B., Nadifi S. (2018). Biomarkers for Alzheimer Disease: Classical and Novel Candidates’ Review. Neuroscience.

[3] Chen G., Xu T., Yan Y., Zhou Y., Jiang Y., Melcher K., Xu H.E. (2017). Amyloid Beta: Structure, Biology and Structure-Based Therapeutic Development. Acta Pharmacol. Sin..

[4] Ossenkoppele R., van der Kant R., Hansson O. (2022). Tau Biomarkers in Alzheimer’s Disease: Towards Implementation in Clinical Practice and Trials. Lancet Neurol..

[5] Wilson D.M., Cookson M.R., Van Den Bosch L., Zetterberg H., Holtzman D.M., Dewachter I. (2023). Hallmarks of Neurodegenerative Diseases. Cell.

[6] Heneka M.T., Carson M.J., El Khoury J., Landreth G.E., Brosseron F., Feinstein D.L., Jacobs A.H., Wyss-Coray T., Vitorica J., Ransohoff R.M. (2015). Neuroinflammation in Alzheimer’s Disease. Lancet Neurol..

[7] Kanashiro A., Hiroki C.H., da Fonseca D.M., Birbrair A., Ferreira R.G., Bassi G.S., Fonseca M.D., Kusuda R., Cebinelli G.C.M., da Silva K.P. (2020). The Role of Neutrophils in Neuro-Immune Modulation. Pharmacol. Res..

[8] Zhang W., Xiao D., Mao Q., Xia H. (2023). Role of Neuroinflammation in Neurodegeneration Development. Signal Transduct. Target. Ther..

[9] Dekens D.W., Eisel U.L.M., Gouweleeuw L., Schoemaker R.G., De Deyn P.P., Naudé P.J.W. (2021). Lipocalin 2 as a Link between Ageing, Risk Factor Conditions and Age-Related Brain Diseases. Ageing Res. Rev..

[10] Garwood C.J., Pooler A.M., Atherton J., Hanger D.P., Noble W. (2011). Astrocytes Are Important Mediators of Aβ-Induced Neurotoxicity and Tau Phosphorylation in Primary Culture. Cell Death Dis..

[11] Carrero I., Gonzalo M.R., Martin B., Sanz-Anquela J.M., Arévalo-Serrano J., Gonzalo-Ruiz A. (2012). Oligomers of β-Amyloid Protein (Aβ1-42) Induce the Activation of Cyclooxygenase-2 in Astrocytes via an Interaction with Interleukin-1β, Tumour Necrosis Factor-α, and a Nuclear Factor κ-B Mechanism in the Rat Brain. Exp. Neurol..

[12] Venegas C., Kumar S., Franklin B.S., Dierkes T., Brinkschulte R., Tejera D., Vieira-Saecker A., Schwartz S., Santarelli F., Kummer M.P. (2017). Microglia-Derived ASC Specks Cross-Seed Amyloid-β in Alzheimer’s Disease. Nature.

[13] Frank-Cannon T.C., Alto L.T., McAlpine F.E., Tansey M.G. (2009). Does Neuroinflammation Fan the Flame in Neurodegenerative Diseases?. Mol. Neurodegener..

[14] Leng F., Edison P. (2021). Neuroinflammation and Microglial Activation in Alzheimer Disease: Where Do We Go from Here?. Nat. Rev. Neurol..

[15] Barron M., Gartlon J., Dawson L.A., Atkinson P.J., Pardon M.C. (2017). A State of Delirium: Deciphering the Effect of Inflammation on Tau Pathology in Alzheimer’s Disease. Exp. Gerontol..

[16] Mander B.A., Dave A., Lui K.K., Sprecher K.E., Berisha D., Chappel-Farley M.G., Chen I.Y., Riedner B.A., Heston M., Suridjan I. (2022). Inflammation, Tau Pathology, and Synaptic Integrity Associated with Sleep Spindles and Memory Prior to β-Amyloid Positivity. Sleep.

[17] Goetz D.H., Holmes M.A., Borregaard N., Bluhm M.E., Raymond K.N., Strong R.K. (2002). The Neutrophil Lipocalin NGAL Is a Bacteriostatic Agent That Interferes with Siderophore-Mediated Iron Acquisition. Mol. Cell.

[18] Romejko K., Markowska M., Niemczyk S. (2023). The Review of Current Knowledge on Neutrophil Gelatinase-Associated Lipocalin (NGAL). Int. J. Mol. Sci..

[19] Zamanian J.L., Xu L., Foo L.C., Nouri N., Zhou L., Giffard R.G., Barres B.A. (2012). Genomic Analysis of Reactive Astrogliosis. J. Neurosci..

[20] Tong J., Huang C., Bi F., Wu Q., Huang B., Liu X., Li F., Zhou H., Xia X.G. (2013). Expression of ALS-Linked TDP-43 Mutant in Astrocytes Causes Non-Cell-Autonomous Motor Neuron Death in Rats. EMBO J..

[21] Law I.K.M., Xu A., Lam K.S.L., Berger T., Mak T.W., Vanhoutte P.M., Liu J.T.C., Sweeney G., Zhou M., Yang B. (2010). Lipocalin-2 Deficiency Attenuates Insulin Resistance Associated With Aging and Obesity. Diabetes.

[22] Behrens V., Voelz C., Müller N., Zhao W., Gasterich N., Clarner T., Beyer C., Zendedel A. (2021). Lipocalin 2 as a Putative Modulator of Local Inflammatory Processes in the Spinal Cord and Component of Organ Cross Talk After Spinal Cord Injury. Mol. Neurobiol..

[23] Dekens D.W., Naudé P.J.W., Engelborghs S., Vermeiren Y., Van Dam D., Oude Voshaar R.C., Eisel U.L.M., De Deyn P.P. (2017). Neutrophil Gelatinase-Associated Lipocalin and Its Receptors in Alzheimer’s Disease (AD) Brain Regions: Differential Findings in AD with and without Depression. J. Alzheimer’s Dis..

[24] Llorens F., Hermann P., Villar-Piqué A., Diaz-Lucena D., Nägga K., Hansson O., Santana I., Schmitz M., Schmidt C., Varges D. (2020). Cerebrospinal Fluid Lipocalin 2 as a Novel Biomarker for the Differential Diagnosis of Vascular Dementia. Nat. Commun..

[25] Li H., Gang Z., Yuling H., Luokun X., Jie X., Hao L., Li W., Chunsong H., Junyan L., Mingshen J. (2006). Different Neurotropic Pathogens Elicit Neurotoxic CCR9- or Neurosupportive CXCR3-Expressing Microglia. J. Immunol..

[26] Cole K.E., Strick C.A., Paradis T.J., Ogborne K.T., Loetscher M., Gladue R.P., Lin W., Boyd J.G., Moser B., Wood D.E. (1998). Interferon–Inducible T Cell Alpha Chemoattractant (I-TAC): A Novel Non-ELR CXC Chemokine with Potent Activity on Activated T Cells through Selective High Affinity Binding to CXCR3. J. Exp. Med..

[27] Gupta N., Lomash V., Rao P.V.L. (2010). Expression Profile of Japanese Encephalitis Virus Induced Neuroinflammation and Its Implication in Disease Severity. J. Clin. Virol..

[28] Lepennetier G., Hracsko Z., Unger M., Van Griensven M., Grummel V., Krumbholz M., Berthele A., Hemmer B., Kowarik M.C. (2019). Cytokine and Immune Cell Profiling in the Cerebrospinal Fluid of Patients with Neuro-Inflammatory Diseases. J. Neuroinflamm..

[29] Fu A., Qiao F., Feng H., Luo Q. (2023). Inhibition of TREM-1 Ameliorates Lipopolysaccharide-Induced Depressive-like Behaviors by Alleviating Neuroinflammation in the PFC via PI3K/Akt Signaling Pathway. Behav. Brain Res..

[30] de Oliveira Matos A., dos Santos Dantas P.H., Figueira Marques Silva-Sales M., Sales-Campos H. (2020). The Role of the Triggering Receptor Expressed on Myeloid Cells-1 (TREM-1) in Non-Bacterial Infections. Crit. Rev. Microbiol..

[31] Zhang C., Kan X., Zhang B., Ni H., Shao J. (2022). The Role of Triggering Receptor Expressed on Myeloid Cells-1 (TREM-1) in Central Nervous System Diseases. Molecular Brain.

[32] Bekris L.M., Khrestian M., Dyne E., Shao Y., Pillai J., Rao S., Bemiller S.M., Lamb B., Fernandez H.H., Leverenz J.B. (2018). Soluble TREM2 and Biomarkers of Central and Peripheral Inflammation in Neurodegenerative Disease. J. Neuroimmunol..

[33] Kulczyńska-Przybik A., Dulewicz M., Doroszkiewicz J., Borawska R., Słowik A., Zetterberg H., Hanrieder J., Blennow K., Mroczko B. (2023). The Relationships between Cerebrospinal Fluid Glial (CXCL12, CX3CL, YKL-40) and Synaptic Biomarkers (Ng, NPTXR) in Early Alzheimer’s Disease. Int. J. Mol. Sci..

[34] Doroszkiewicz J., Kulczyńska-Przybik A., Dulewicz M., Borawska R., Zajkowska M., Słowik A., Mroczko B. (2023). Potential Utility of Cerebrospinal Fluid Glycoprotein Nonmetastatic Melanoma Protein B as a Neuroinflammatory Diagnostic Biomarker in Mild Cognitive Impairment and Alzheimer’s Disease. J. Clin. Med..

[35] Raivich G., Bohatschek M., Kloss C.U.A., Werner A., Jones L.L., Kreutzberg G.W. (1999). Neuroglial Activation Repertoire in the Injured Brain: Graded Response, Molecular Mechanisms and Cues to Physiological Function. Brain Res. Rev..

[36] Choi J., Lee H.W., Suk K. (2011). Increased Plasma Levels of Lipocalin 2 in Mild Cognitive Impairment. J. Neurol. Sci..

[37] Zhao Y., Li T., Jiang Z., Gai C., Yu S., Xin D., Li T., Liu D., Wang Z. (2024). The MiR-9-5p/CXCL11 Pathway Is a Key Target of Hydrogen Sulfide-Mediated Inhibition of Neuroinflammation in Hypoxic Ischemic Brain Injury. Neural Regen. Res..

[38] das Neves S.P., Taipa R., Marques F., Soares Costa P., Monárrez-Espino J., Palha J.A., Kivipelto M. (2021). Association Between Iron-Related Protein Lipocalin 2 and Cognitive Impairment in Cerebrospinal Fluid and Serum. Front. Aging Neurosci..

[39] Naudé P.J.W., Nyakas C., Eiden L.E., Ait-Ali D., van der Heide R., Engelborghs S., Luiten P.G.M., De Deyn P.P., den Boer J.A., Eisel U.L.M. (2012). Lipocalin 2: Novel Component of Proinflammatory Signaling in Alzheimer’s Disease. FASEB J..

[40] Rosén C., Mattsson N., Johansson P.M., Andreasson U., Wallin A., Hansson O., Johansson J.O., Lamont J., Svensson J., Blennow K. (2011). Discriminatory Analysis of Biochip-Derived Protein Patterns in CSF and Plasma in Neurodegenerative Diseases. Front. Aging Neurosci..

[41] Li X., Wang X., Guo L., Wu K., Wang L., Rao L., Liu X., Kang C., Jiang B., Li Q. (2023). Association between Lipocalin-2 and Mild Cognitive Impairment or Dementia: A Systematic Review and Meta-Analysis of Population-Based Evidence. Ageing Res. Rev..

[42] Bi F., Huang C., Tong J., Qiu G., Huang B., Wu Q., Li F., Xu Z., Bowser R., Xia X.G. (2013). Reactive Astrocytes Secrete Lcn2 to Promote Neuron Death. Proc. Natl. Acad. Sci. USA.

[43] Marques F., Mesquita S.D., Sousa J.C., Coppola G., Gao F., Geschwind D.H., Columba-Cabezas S., Aloisi F., Degn M., Cerqueira J.J. (2012). Lipocalin 2 Is Present in the EAE Brain and Is Modulated by Natalizumab. Front. Cell Neurosci..

[44] Ferreira A.C., Dá Mesquita S., Sousa J.C., Correia-Neves M., Sousa N., Palha J.A., Marques F. (2015). From the Periphery to the Brain: Lipocalin-2, a Friend or Foe?. Prog. Neurobiol..

[45] Jang E., Kim J.-H., Lee S., Kim J.-H., Seo J.-W., Jin M., Lee M.-G., Jang I.-S., Lee W.-H., Suk K. (2013). Phenotypic Polarization of Activated Astrocytes: The Critical Role of Lipocalin-2 in the Classical Inflammatory Activation of Astrocytes. J. Immunol..

[46] Mesquita S.D., Ferreira A.C., Falcao A.M., Sousa J.C., Oliveira T.G., Correia-Neves M., Sousa N., Marques F., Palha J.A. (2014). Lipocalin 2 Modulates the Cellular Response to Amyloid Beta. Cell Death Differ..

[47] Kjeldsen L., Johnsen A.H., Sengeløv H., Borregaard N. (1993). Isolation and Primary Structure of NGAL, a Novel Protein Associated with Human Neutrophil Gelatinase. J. Biol. Chem..

[48] Marques F., Rodrigues A.J., Sousa J.C., Coppola G., Geschwind D.H., Sousa N., Correia-Neves M., Palha J.A. (2008). Lipocalin 2 Is a Choroid Plexus Acute-Phase Protein. J. Cereb. Blood Flow Metab..

[49] Naudé P.J.W., Mommersteeg P.M.C., Zijlstra W.P., Gouweleeuw L., Kupper N., Eisel U.L.M., Kop W.J., Schoemaker R.G. (2014). Neutrophil Gelatinase-Associated Lipocalin and Depression in Patients with Chronic Heart Failure. Brain Behav. Immun..

[50] Ferreira A.C., Pinto V., Dá Mesquita S., Novais A., Sousa J.C., Correia-Neves M., Sousa N., Palha J.A., Marques F. (2013). Lipocalin-2 Is Involved in Emotional Behaviors and Cognitive Function. Front. Cell Neurosci..

[51] Flo T.H., Smith K.D., Sato S., Rodriguez D.J., Holmes M.A., Strong R.K., Akira S., Aderem A. (2004). Lipocalin 2 Mediates an Innate Immune Response to Bacterial Infection by Sequestrating Iron. Nature.

[52] Naudé P.J.W., Ramakers I.H.G.B., van der Flier W.M., Jiskoot L.C., Reesink F.E., Claassen J.A.H.R., Koek H.L., Eisel U.L.M., De Deyn P.P. (2021). Serum and Cerebrospinal Fluid Neutrophil Gelatinase-Associated Lipocalin (NGAL) Levels as Biomarkers for the Conversion from Mild Cognitive Impairment to Alzheimer’s Disease Dementia. Neurobiol. Aging.

[53] Balusu S., Brkic M., Libert C., Vandenbroucke R.E. (2016). The Choroid Plexus-Cerebrospinal Fluid Interface in Alzheimer’s Disease: More than Just a Barrier. Neural Regen. Res..

[54] Suárez-Calvet M., Caballero M.Á.A., Kleinberger G., Bateman R.J., Fagan A.M., Morris J.C., Levin J., Danek A., Ewers M., Haass C. (2016). Early Changes in CSF STREM2 in Dominantly Inherited Alzheimer’s Disease Occur after Amyloid Deposition and Neuronal Injury. Sci. Transl. Med..

[55] Bemiller S.M., McCray T.J., Allan K., Formica S.V., Xu G., Wilson G., Kokiko-Cochran O.N., Crish S.D., Lasagna-Reeves C.A., Ransohoff R.M. (2017). TREM2 Deficiency Exacerbates Tau Pathology through Dysregulated Kinase Signaling in a Mouse Model of Tauopathy. Mol. Neurodegener..

[56] Piccio L., Deming Y., Del-Águila J.L., Ghezzi L., Holtzman D.M., Fagan A.M., Fenoglio C., Galimberti D., Borroni B., Cruchaga C. (2016). Cerebrospinal Fluid Soluble TREM2 Is Higher in Alzheimer Disease and Associated with Mutation Status. Acta Neuropathol..

[57] Zhang X., Tang L., Yang J., Meng L., Chen J., Zhou L., Wang J., Xiong M., Zhang Z. (2023). Soluble TREM2 Ameliorates Tau Phosphorylation and Cognitive Deficits through Activating Transgelin-2 in Alzheimer’s Disease. Nat. Commun..

[58] McKhann G.M., Knopman D.S., Chertkow H., Hyman B.T., Jack C.R., Kawas C.H., Klunk W.E., Koroshetz W.J., Manly J.J., Mayeux R. (2011). The Diagnosis of Dementia Due to Alzheimer’s Disease: Recommendations from the National Institute on Aging-Alzheimer’s Association Workgroups on Diagnostic Guidelines for Alzheimer’s Disease. Alzheimers Dement..

[59] Albert M.S., DeKosky S.T., Dickson D., Dubois B., Feldman H.H., Fox N.C., Gamst A., Holtzman D.M., Jagust W.J., Petersen R.C. (2011). The Diagnosis of Mild Cognitive Impairment Due to Alzheimer’s Disease: Recommendations from the National Institute on Aging-Alzheimer’s Association Workgroups on Diagnostic Guidelines for Alzheimer’s Disease. Alzheimers Dement..

