# Supplementary material for: Associations between Microglia and Astrocytic Proteins and Tau Biomarkers across the Continuum of Alzheimer’s Disease
Source: Int J Mol Sci. 2024 Jul 9;25(14):7543. doi: 10.3390/ijms25147543 (PMC11277045; doi:10.3390/ijms25147543)
Supplement: Supplementary file 1 [file ijms-25-07543-s001.zip › ijms-3055816-supplementary.pdf]

Suplementarny Materials

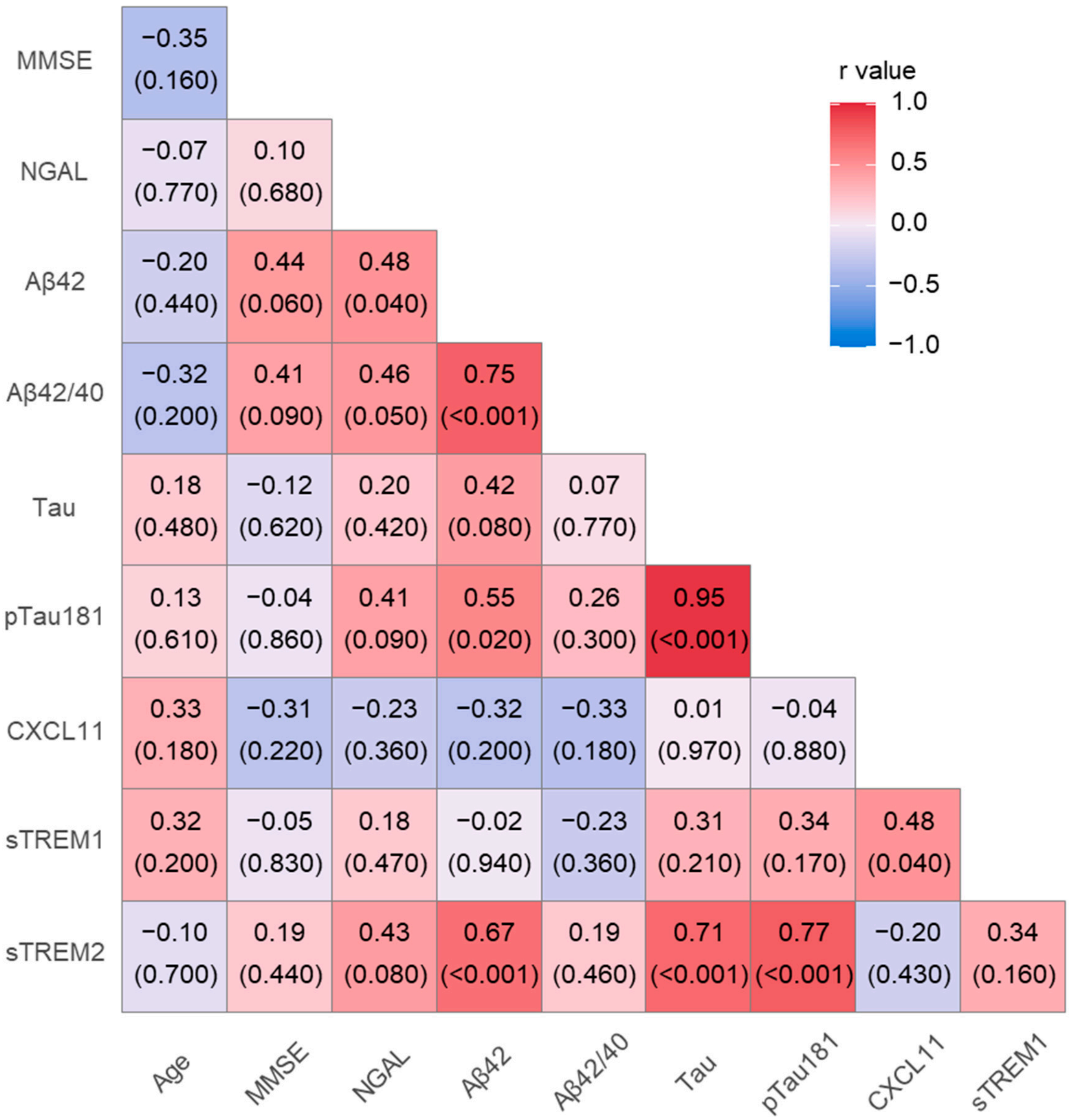

Figure S1. Cerebrospinal fluid levels of pro and anti-inflammatory in MCI group. NGAL – Neutrophil gelatinase-associated lipocalin. NGAL – Neutrophil gelatinase-associated lipocalin, ; sTREM1 - soluble triggering receptor expressed on myeloid cells-1; sTREM2 - soluble triggering receptor expressed on myeloid cells-2; *** - p < 0.001

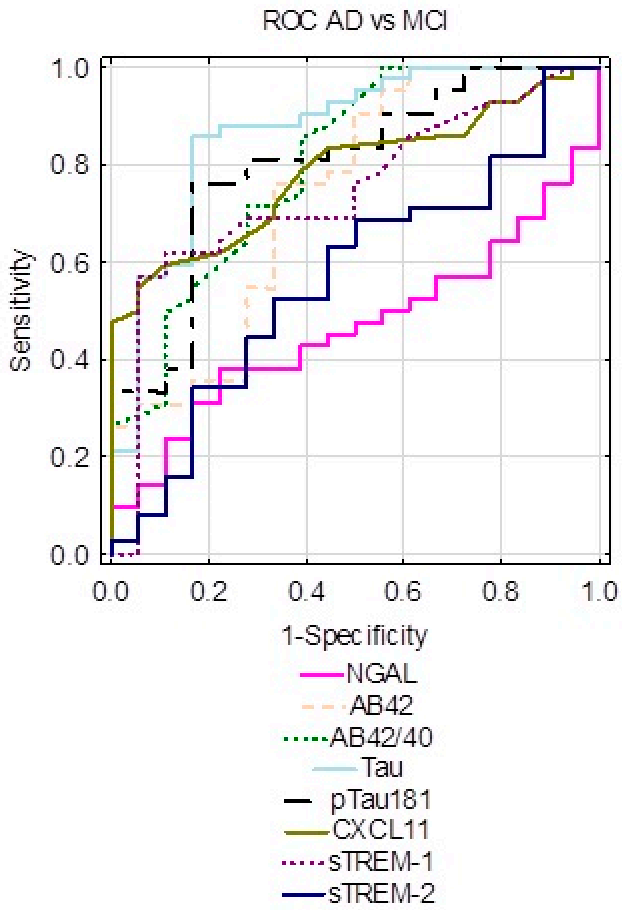

Figure S2. Comparison of area under ROC curves (AUC) for cerebrospinal fluid levels of pro- and anti-inflammatory and classical AD biomarkers in MCI and CTRL groups. NGAL – Neutrophil gelatinase-associated lipocalin, ; sTREM1 - soluble triggering receptor expressed on myeloid cells-1; sTREM2 - soluble triggering receptor expressed on myeloid cells-2;
